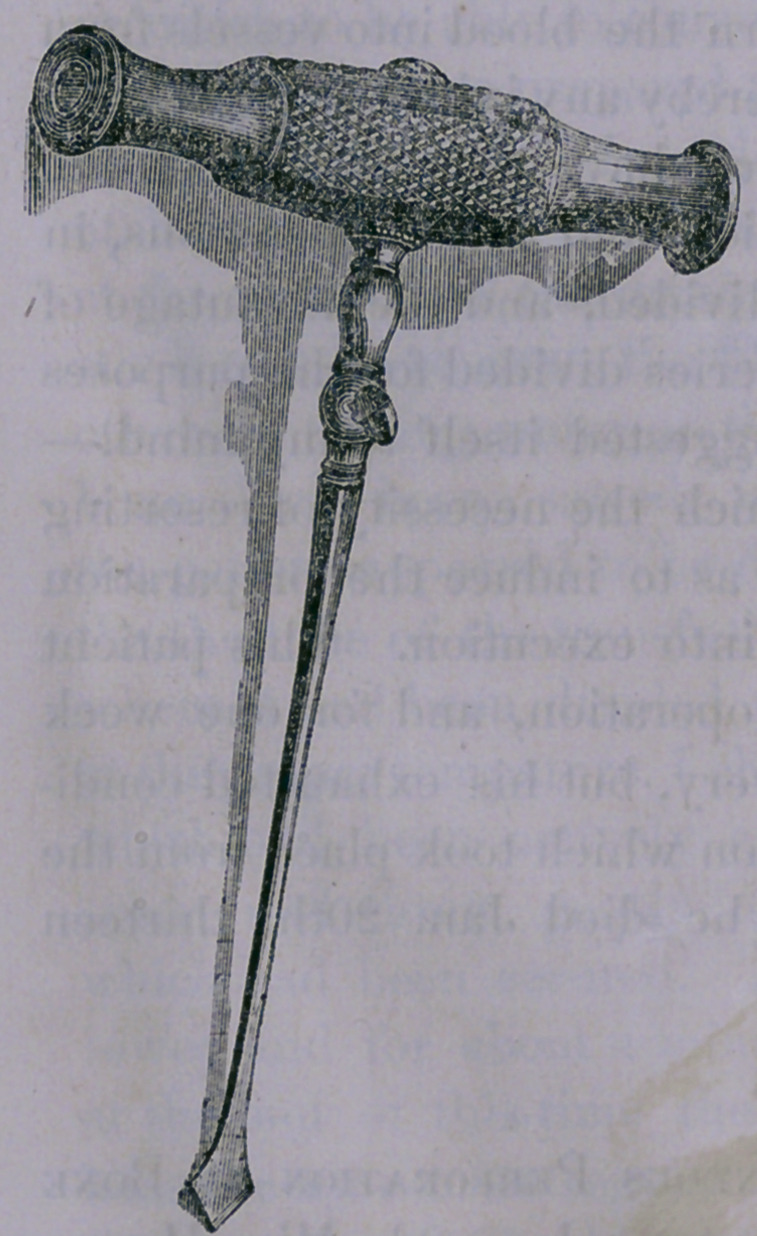# On the Use of Subcataneous Perforation of Bone in Anchylosis of the Knee Joint

**Published:** 1860-02

**Authors:** 


3. On the use of Subcataneous Perforation of Bone in Anchylosis of the Knee Joint.—Jan. 2d, Miss House, æt. 16 years, from Kankakee, Ill., presented herself at the clinic for the purpose of being treated for an anchylosis of the left knee. The leg was flexed to about 60 degrees from the straight position, and capable of movement just percepible by the application of force. The mother stated that two years previously, the knee was attacked by a severe pain, which was followed by swelling of the member from the foot to hip. The pain continued for several months and was regarded as rheumatic.

At present the health is good, and the knee free from pain, swelling, and tenderness.

*Treatment.*—Jan. 4th, 1860, the patient being under the influence of chloroform, efforts were made to break up the adhesions and flex the leg upon the thigh. Considerable movement of the joint was produced, but the patella seemed firmly fixed to the femur and could not be moved by any force deemed proper to apply.

In order to detach it, the instrument used for subcataneous division of bone, heretofore described in this *Journal*, (see fig. half size) was insérted through the skin on the outside of the patella, and carried between the two bones. At the upper part only, the patella was fixed to the femur by bone. Separation was effected by perforation and by using the instrument as a lever, and the patella was loosened with a snap. The instrument was then withdrawn, the puncture covered with adhesive plaster, compress and roller. The skin was drawn forward the puncture, so that after the wiihdrawal of the instrument, the wound was distant from the external margin of the patella one and a half inches.

Some appearance of ecchymosis followed, but little tenderness. Ten days after, viz: January 13th, 1860, the puncture was healed. The patient was again put under the influence of chloroform and efforts at flexion repeated. This time they were successful, and with moderate force the leg was bent beyond a right angle and considerably straightened; the patella following the movements. The pain following this treatment was not great. An extending apparatus was applied on the 18th, and gradual extension kept up. At the present time the leg is nearly in a straight position.

This method of detaching the patella from the femur, has not been used before, and is an application of the plan proposed by tho author in the prize essay of the American Medical Association for 1854. It has, however, been used three times in other deformities; viz: first by the author for deformity after fracture of the leg; second, by Professor Paul F. Eve for the same cause; third, by Professor Pancoast for anchylosis of the knee. The result in the first two cases was perfectly successful; that of Professor Pancoast was progressing favorably, when the report was made.

## Figures and Tables

**Figure f1:**